# Hyper secretion of *Thermobifida fusca* β-glucosidase via a Tat-dependent signal peptide using *Streptomyces lividans*

**DOI:** 10.1186/1475-2859-12-88

**Published:** 2013-10-01

**Authors:** Takaya Miyazaki, Shuhei Noda, Tsutomu Tanaka, Akihiko Kondo

**Affiliations:** 1 Department of Chemical Science and Engineering, Graduate School of Engineering, Kobe University, 1-1 Rokkodai, Nada, Kobe 657-8501, Japan; 2 Biomass Engineering Program, RIKEN, 1-7-22 Suehiro-cho, Tsurumi-ku, Yokohama, Kanagawa 230-0045, Japan; 3 Department of Food Bioscience and Technology, College of Life Sciences and Biotechnology, Korea University, Seoul 136-713, Republic of Korea

**Keywords:** *Streptomyces*, Protein secretion, Signal peptide sequence, Tat pathway

## Abstract

**Background:**

Protein production as secretory-form is a powerful tool in industrial enzyme production due to the simple purification procedure. *Streptomyces lividans* is a versatile host for secretory production of useful proteins. In order to expand the amount of secreted protein, signal peptide sequences, which encourage protein secretion from inside cell to extracellular environment, are one of the most significant factors. In this study, we focused on *Streptomyces lividans* as a host strain to secrete useful proteins, and screened for signal peptides from the biomass-degradation enzymes derived from *Thermobifida fusca* YX and *S. lividans*.

**Results:**

Three candidate signal peptides were isolated and evaluated for their protein secretion ability using β-glucosidase derived from *T. fusca* YX, which is a non-secreted protein, as a model protein. Using *S. lividans* xylanase C signal peptide, the amount of produced the β-glucosidase reached 10 times as much as that when using *Streptomyces cinnamoneus* phospholipase D signal peptide, which was identified as a versatile signal peptide in our previous report. In addition, the introduction of the β-glucosidase fused to xylanase C signal peptide using two kinds of plasmid, pUC702 and pTYM18, led to further protein secretion, and the maximal level of produced the β-glucosidase increased up to 17 times (1.1 g/l) compared to using only pUC702 carrying the β-glucosidase fused to *S. cinnamoneus* phospholipase D signal peptide.

**Conclusion:**

In the present study, we focused on signal peptide sequences derived from biomass degradation enzymes, which are usually secreted into the culture supernatant, and screened for signal peptides leading to effective protein secretion. Using the signal peptides, the hyper-protein secretion system was successfully demonstrated for the cytoplasmic β-glucosidase.

## Background

The production of useful proteins using microbes has attracted attention due to scientific, therapeutic, environmental, and agricultural applications. The secretory production of proteins has three major potential advantages: secreted target proteins are usually natively folded, the degradation of target proteins by intracellular proteases can be suppressed, and there is a reduced requirement for expensive extraction and purification procedures [1-4]. Therefore, the secretory production of valuable proteins is an industrially effective method to simplify purification procedures and avoid refolding processes and extraction from cells. In the last few decades, many protein expression systems have been developed using various kinds of microbes. Park et al. succeeded in the production of FDA approved pharmaceuticals such as insulin and hepatitis B surface antigen using *Saccharomyces cerevisiae*[5], and *Bacillus subtilis* has been used to successfully produce esterase and cutinase [6]. Among microbial systems, *Escherichia coli* is the most widely used due to its high expression levels (1–5 g/l) and simplicity of genetic manipulation [7-9]. However, with *E. coli*, produced proteins are usually obtained in the intracellular fraction and need to be extracted from cells.

*Streptomyces* species are Gram-positive, aerobic, mycelia-forming oil bacteria. In general, *Streptomyces* are widely used as a host to produce antibiotics and other industrial enzymes [10-12]. In particular, *Streptomyces lividans* is an attractive host that has high ability to secrete heterologous proteins in the culture supernatant. Sianidis et al. successfully produced xyloglucanase from *Jonesia sp.*[13]*.* Lin et al. successfully produced *Streptomyces platensis* transglutaminase using *S. lividans* as the expression host [14]. We also reported the secretory production of two cellulases and transglutaminase using a secretory system that consisted of a putative promoter (*pld* promoter) and terminator regions (*pld* terminator), and the signal peptide sequence (Plds) derived from the phospholipase D gene of *Streptomyces cinnamoneus*[15].

Most secretory proteins contain an N-terminal signal peptide that is cleaved by a membrane-bound signal peptidase. Signal peptides play important roles in encouraging protein secretion and have three domains, a positively charged N-domain, a hydrophobic H-domain, and a C-domain containing the three amino acids that form the signal peptidase recognition site [1,12]. Various kinds of secretory proteins in bacteria are exported across the cytoplasmic membrane by the Sec system, which acts on unfolded polypeptides chains. There are a number of reports concerning protein secretion using signal peptides dependent on the Sec system [1,12], including Plds [15]. However, a novel alternate translocation system, the twin-arginine-dependent translocation (Tat) system, was recently identified [16-18]. The Tat system can transport fully-folded proteins across the membrane, and the function and mechanism of the system have been widely researched in various bacteria [16-18].

In order to enhance the secretion of heterologous proteins, various approaches have been recently tried. These include the identification of inducible promoters to increase gene transcript levels, and the overexpression of components controlling protein secretion [19]. Overexpression of the Tat pathway component, TatABC, encourages increased yields of up to 5–30 times of secreted proteins dependent on the Tat system in *Streptomyces* and *Corynebacterium glutamicum*[19,20]. In a report of protein secretion using *B. subtilis*, screening for novel signal peptides was carried out in order to enhance the amount of secreted protein [21]. However, the yield of secreted proteins was relatively low compared to that of intracellular protein production using *E. coli.*

In the present study, we constructed a hyper-secretion system for useful proteins by focusing on the signal peptide to enhance protein secretion and on the copy number of target genes to increase protein produced by *S. lividans*. We hypothesized that biomass degradation enzymes, which are generally highly secreted, involve signal peptides to encourage effective secretion. We first investigated the secretion levels of several kinds of biomass degradation enzymes derived from *S. lividans* and *Thermobifida fusca* YX using *S. lividans* as a host. *T. fusca* is an aerobic, thermophilic, filamentous soil bacterium that is a major degrader of plant cell walls [22], and some proteins derived from *T. fusca* have been successfully expressed using *S. lividans*[15]. Among the biomass degradation enzymes successfully secreted from *S. lividans*, 3 different signal peptides (derived from *S. lividans* xylanase C, *T. fusca* β-1,4-exocellulase, and *T. fusca* xylosidase) dependent on the Tat system were successfully obtained. We used β-glucosidase from *T. fusca* YX (BGL) as a model heterologous protein, which is a non-secreted protein. We focused on the copy number of BGL genes in order to achieve further protein secretion, and introduced a gene encoding BGL fused to the xylanase C signal peptide (XCs) into *S. lividans* using a multi-copy vector and an integrative vector, pUC702 and pTYM18 [15,23]. Using this strategy, the amount of secreted BGL was drastically improved compared to that when using Plds that we previously isolated [15]. We successfully constructed a hyper-secretion system for useful proteins, and our system is more efficiently than those from previous reports concerning protein secretion using *Streptomyces*.

## Results

### Construction of biomass-degradation enzyme library derived from *Streptomyces lividans* and *Thermobifida fusca* YX

In this study, as an approach to obtain novel signal peptides encouraging efficient protein secretion, we focused on biomass degradation enzymes. We hypothesized that biomass degradation enzymes involve signal peptides because they are highly secreted, and we carried out the secretory production of 25 kinds of biomass-degradation enzymes derived from *S. lividans* and *T. fusca* YX in *S. lividans.* The genes encoding each enzyme were introduced downstream of the phospholipase D promoter region (*pld* promoter) in the multi-copy type vector pUC702. After transformation and cultivation for 3 days, the supernatant of each transformant was analyzed by western blotting using primary rabbit anti-(His)_6_ and secondary goat anti-rabbit immunoglobulin G alkaline phosphatase conjugated antibodies, and we evaluated the amount of each protein (Table 1). As a result, we successfully obtained 17 kinds of biomass-degradation enzymes secreted in *S. lividans*. Thus, we constructed a biomass-degradation enzymes library including both Sec-type and Tat-type signal peptides, and the signal peptides involved in these enzymes were considered as candidates to encourage protein secretion.

**Table 1:** **Biomass degradation enzyme library from *S. lividans *and *T. fusca *YX**[22]

**Sec-dependent signal peptides**			**Tat-dependent signal peptides**		
	**Protein**	**Expression**		**Protein**	**Expression**
SlvXlnA	Xylanase	+	SlvXlnC	Endo-xylanase	+
SlvXlnB	Endo-xylanase	+	Tfu0082	Acetyl xylan esterase	+
SlvCelB	Endo-glucanase	+	Tfu0153	Pectate-lyase	+
Tfu2791	Endo-xylanase	+	Tfu0620	β-1,4-exocellulase	+
Tfu1268	Cellulose-binding protein	+	Tfu0868	Exochitinase	-
Tfu1616	Xylanase/Galactosidase	-	Tfu0900	β -Mannanase	+
Tfu1621	Acetyl xylan esterase	-	Tfu0985	Amylase	+
Tfu1629	Glucosidase	+	Tfu1213	Endo-xylanase	+
Tfu1665	Cellulose-binding protein	+	Tfu2168	Pectate-lyase	+
Tfu2789	Acetyl xylan esterase	-	Tfu2486	Xylosidase	+
Tfu2990	Acetyl xylan esterase	-	Tfu2712	Cellulase	-
			Tfu2788	Acetyl xylan esterase	+
			Tfu1612	Xyloglucanase	+
			Tfu2176	Cellulase	+

### Screening for signal peptides encouraging protein secretion using a model protein

**Figure 1:**
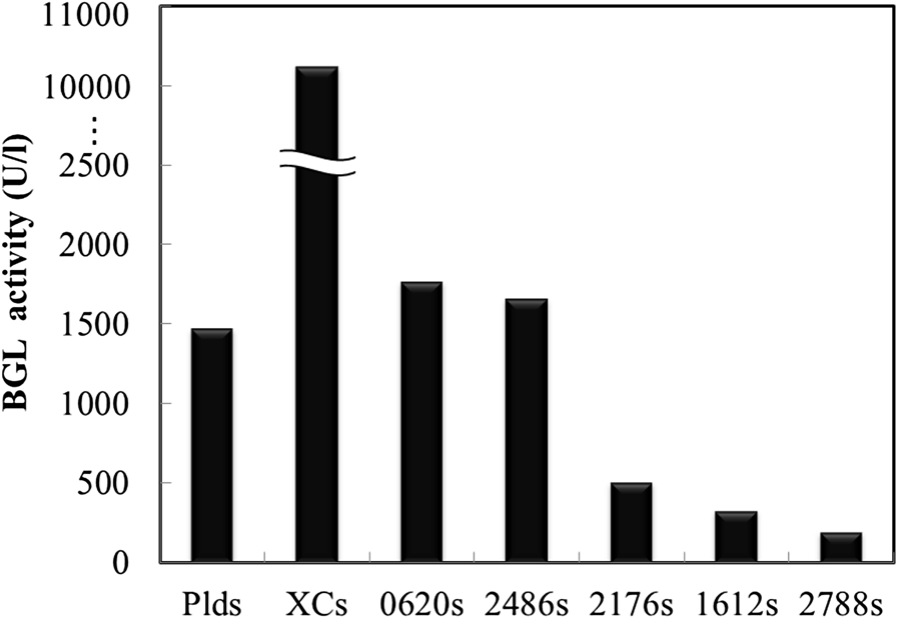
BGL activity detected in the culture supernatant of each transformant cultured using test tubes after 3 days.

The signal peptides of biomass-degradation enzymes secreted in *S. lividans* were evaluated using β-glucosidase from *T. fusca* YX (BGL) as a model heterologous protein. 17 kinds of signal peptides were isolated from 25 kinds of biomass-degradation enzymes and fused upstream of the gene encoding BGL (Tfu0937), and we constructed each vector for BGL secretion (Table 2). After transformation, we evaluated the amount of BGL each transformant produced with pre-cultivation using a test tube. The activity present in the supernatant of the top 7 signal peptides encouraging BGL production, after 3 days cultivation, is shown in Figure 1. The signal peptide derived from *S. cinnamoneus* phospholipase D (Plds), previously described by our group, is included [15]. For 3 of the signal peptides (XCs, Tfu0620s, Tfu2486s), the amount of secreted BGL was higher than that when using Plds. The sequence of each signal peptide is shown in Table 3. Here, we represented the top 7 signal peptides encouraging BGL production (including Plds) in Figure 1.

**Table 2:** Strains, plasmids, transformants, and oligonucleotide primers used in this study

**Strain, plasmid, primer, or transformant**	**Relevant features**	**Source or reference**
Strain		
*Escherichia coli* strains		
Nova blue	*endA1 hsdR17*(r_*K12*_^*-*^m_*K12*_^+^) *supE44 thi-I gyrA96 relA1 lac*	Novagene
	recA1/F’[proAB + lacIq ZΔM15::Tn10(Tetr)]	
*Streptomyces lividans* 1326	WT strain	NBRC
Plasmids		
pUC702-pro-sig-term	Versatile vector for protein expression; thiostrepton resistance marker	[15]
pUC702-plds-bgl	Vector for secreting β-glucosidase(Tfu0937) using plds; thiostrepton resistance marker	[15]
pUC702-xcs-bgl	Vector for secreting β-glucosidase(Tfu0937) using xcs; thiostrepton resistance marker	This study
pCU702-tfu0620s-bgl	Vector for secreting β-glucosidase(Tfu0937) using tfu0620s; thiostrepton resistance marker	This study
pUC702-tfu2486s-bgl	Vector for secreting β-glucosidase(Tfu0937) using tfu2486s; thiostrepton resistance marker	This study
pUC702-tfu2176s-bgl	Vector for secreting β-glucosidase(Tfu0937) using tfu2716s; thiostrepton resistance marker	This study
pUC702-tfu1612s-bgl	Vector for secreting β-glucosidase(Tfu0937) using tfu1612s; thiostrepton resistance marker	This study
pUC702-tfu2788s-bgl	Vector for secreting β-glucosidase(Tfu0937) using tfu2788s; thiostrepton resistance marker	This study
pTYM18	Intergeneric conjugation vector; kanamycin resistance marker	[23]
pTYM18-xcs-bgl	Vector for secreting β-glucosidase(Tfu0937) using xcs; kanamycin resistance marker	This study
pTYM18-plds-bgl	Vector for secreting β-glucosidase(Tfu0937) using xcs; kanamycin resistance marker	This study
Transformants		
*S. lividans*/pU-plds-bgl	Transformant harboring pUC702-plds-β-glucosidase (Tfu0937)	[15]
*S. lividans*/pU-xcs-bgl	Transformant harboring pUC702-xcs-β-glucosidase (Tfu0937)	This study
*S. lividans*/pU-0620 s-bgl	Transformant harboring pUC702-tfu0620s-β-glucosidase (Tfu0937)	This study
*S. lividans*/pU-2486 s-bgl	Transformant harboring pUC702-tfu2486s-β-glucosidase (Tfu0937)	This study
*S. lividans*/pU-2176 s-bgl	Transformant harboring pUC702-tfu2176s-β-glucosidase (Tfu0937)	This study
*S. lividans*/pU-1612 s-bgl	Transformant harboring pUC702-tfu1612s-β-glucosidase (Tfu0937)	This study
*S. lividans*/pU-2788 s-bgl	Transformant harboring pUC702-tfu2788s-β-glucosidase (Tfu0937)	This study
*S. lividans*/pT-xcs-bgl	Transformant integrated pTYM18-xcs-β-glucosidase (Tfu0937)	This study
*S. lividans*/pTxcs-pUxcs	Transformant integrated pTYM18-xcs-β-glucosidase (Tfu0937) and harboring pUC702-xcs-β-glucosidase (Tfu0937)	This study
*S. lividans*/pTxcs-pUplds	Transformant integrated pTYM18-xcs-β-glucosidase (Tfu0937) and harboring pUC702-plds-β-glucosidase (Tfu0937)	This study
Oligonucleotide primers		
xcs_Fw	aGCATGCatgcagcaggacggcacacagcagg	
xcs_Rv	tttGCTAGCggcgtgggctgtgccgggcagcagc	
tfu0620s_Fw	TCGTTTAAGGATGCAatgagtaaagttcgtgccacgaaca	
tfu0620s_Rv	CGATTGCGAGGTCACggcggcgttggccggagcagcgaac	
tfu2486s_Fw	TCGTTTAAGGATGCAatgtttcgacgtctgcctgtgctgg	
tfu2486s_Rv	CGATTGCGAGGTCACtgccgcctgagcgtccacgtcagcg	
tfu2176s_Fw	TCGTTTAAGGATGCAatgtccgtcactgaacctcctcccc	
tfu2176s_Rv	CGATTGCGAGGTCACttcggcgtgggcggttcccgtggcc	
tfu1612s_Fw	TCGTTTAAGGATGCAatgacagcaacagcacagcgaacac	
tfu1612s_Rv	CGATTGCGAGGTCACggcggcagcggagtggacgaggccg	
Tfu2788s_Fw	TCGTTTAAGGATGCAatgtccgtcactgaacctcctcccc	
Tfu2788s_Rv	CGATTGCGAGGTCACttcggcgtgggcggttcccgtggcc	

**Table 3:** Signal peptide sequence identified in each enzyme

**Signal name**	**Secretion system**	**Amino acids sequences**
Plds	Sec	MLRHRLRRLH RLTRSAAVSAVVLAALPAAPAFAS
XCs	Tat	MQQDGTQQDRIKQSPAPLNGMSRRGFLGGAGTLALATASGLLLPGTAHA
Tfu0620s	Tat	MSKVRATNRRSWMRRGLAAASGLALGASMVAFAAPANAA
Tfu2486s	Tat	MFRRLPVLAGATVLLFTTACGGGSAPRPGERTTQISDPADVDAQAA
Tfu2176s	Tat	MSVTEPPPRRRGRHSRARRFLTSLGATAALTAGMLGVPLATGTAHAE
Tfu1612s	Tat	MTATAQRTPPPPTPRRRGIIARALTCIAAAATVAAVGLVHSAAA
Tfu2788s	Tat	MTTVPRRSPLRKRLLVALCALGLAFTSAATAHAQ

**Figure 2:**
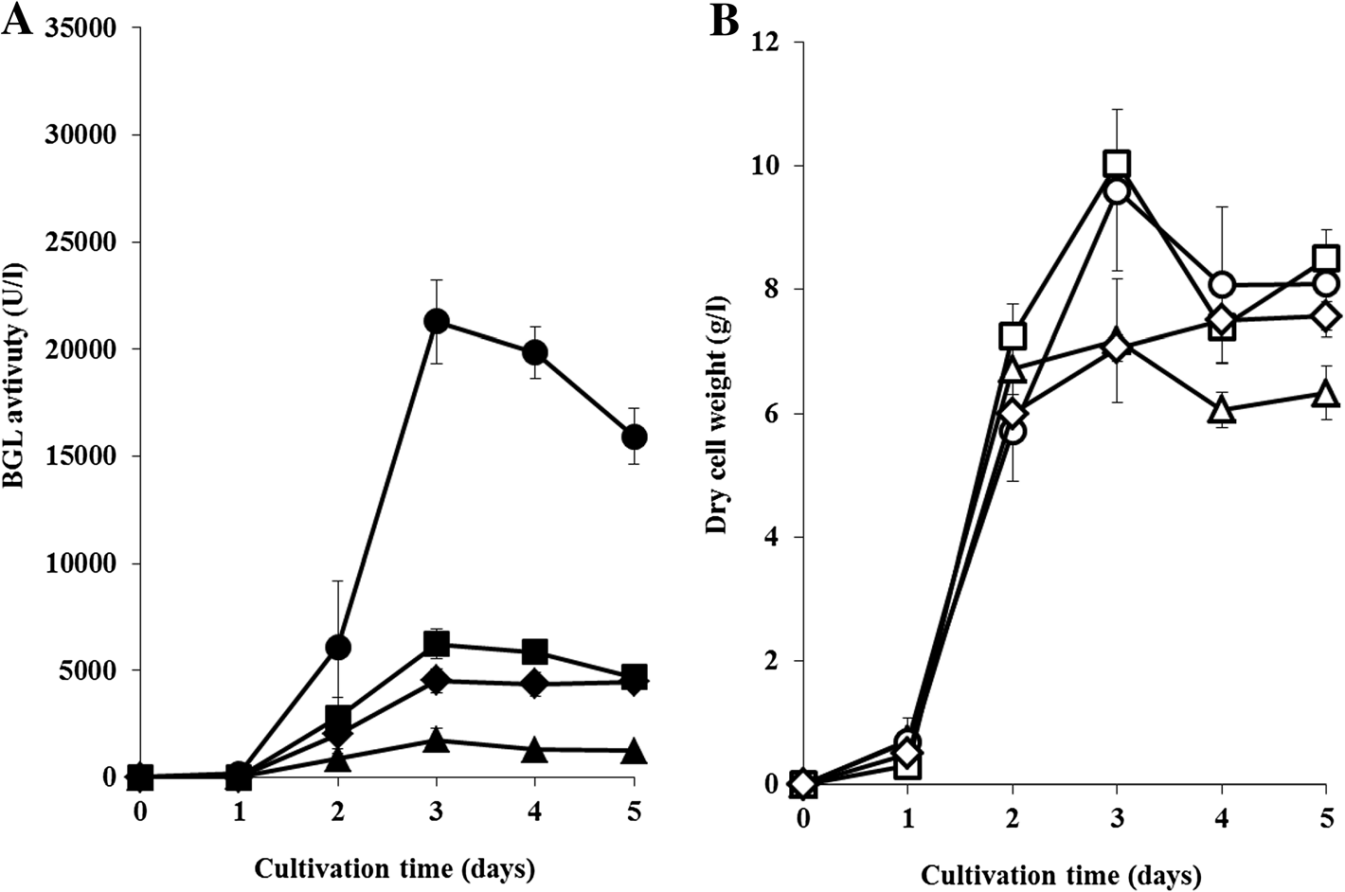
**The secretory production of BGL using signal peptides screened in this study. (A)** Time-course of culture supernatant β-glucosidase activity: *S. lividans*/pU-xcs-bgl (closed circles); *S. lividans*/pU-0620 s-bgl (closed squares); *S. lividans*/pU-2486 s-bgl (closed diamonds); *S. lividans*/pU-plds-bgl (closed triangles) in modified TSB medium with 5% tryptone and 3% glucose. Each data point shows the average of 3 independent experiments, and error bars represent the standard deviation. **(B)** Time-course of dry cell weight: *S. lividans*/pU-xcs-bgl (open circles); *S. lividans*/pU-0620 s-bgl (open squares); *S. lividans*/pU-2486 s-bgl (open diamonds); *S. lividans*/pU-plds-bgl (open triangles) in modified TSB medium with 5% tryptone and 3% glucose. Each data point shows the average of 3 independent experiments, and error bars represent the standard deviation.

In order to investigate in detail BGL productivity, *S. lividans*/pU-xcs-bgl, *S. lividans*/pU-tfu0620-bgl, *S. lividans*/pU-tfu2486-bgl, and *S. lividans*/pU-plds-bgl were cultured in 100 ml of the modified TSB medium. Figure 2(A) shows the time-courses of BGL activity of each transformant. The maximal level of BGL activity reached 21,000 U/l in the cultivation of *S. lividans*/pU-xcs-bgl, which was 10 times as much as that of *S. lividans*/pU-plds-bgl. BGL activity in the culture supernatant of *S. lividans*/pU-plds-bgl was about 1800 U/l. In addition, 645 mg/l of BGL was estimated in the culture supernatant of *S. lividans*/pU-xcs-bgl after 3 days cultivation. In the case of *S. lividans*/pU-tfu0620-bgl or *S. lividans*/pU-tfu2486-bgl, BGL activity reached 6,200 or 4,500 U/l, which was 3.5 or 2.6 times as much as that of *S. lividans*/pU-plds-bgl (Figure 2(A)). Figure 2(B) shows the time-courses of dry cell weight of each BGL-secreting transformant. The cell growth of *S. lividans*/pU-xcs-bgl, *S. lividans*/pU-tfu0620-bgl, *S. lividans*/pU-tfu2486-bgl, and *S. lividans*/pU-plds-bgl were similar, whereas there was great difference in BGL activity among each transformant.

### Hyper-secretion of BGL in *S. lividans* using two different plasmids

**Figure 3:**
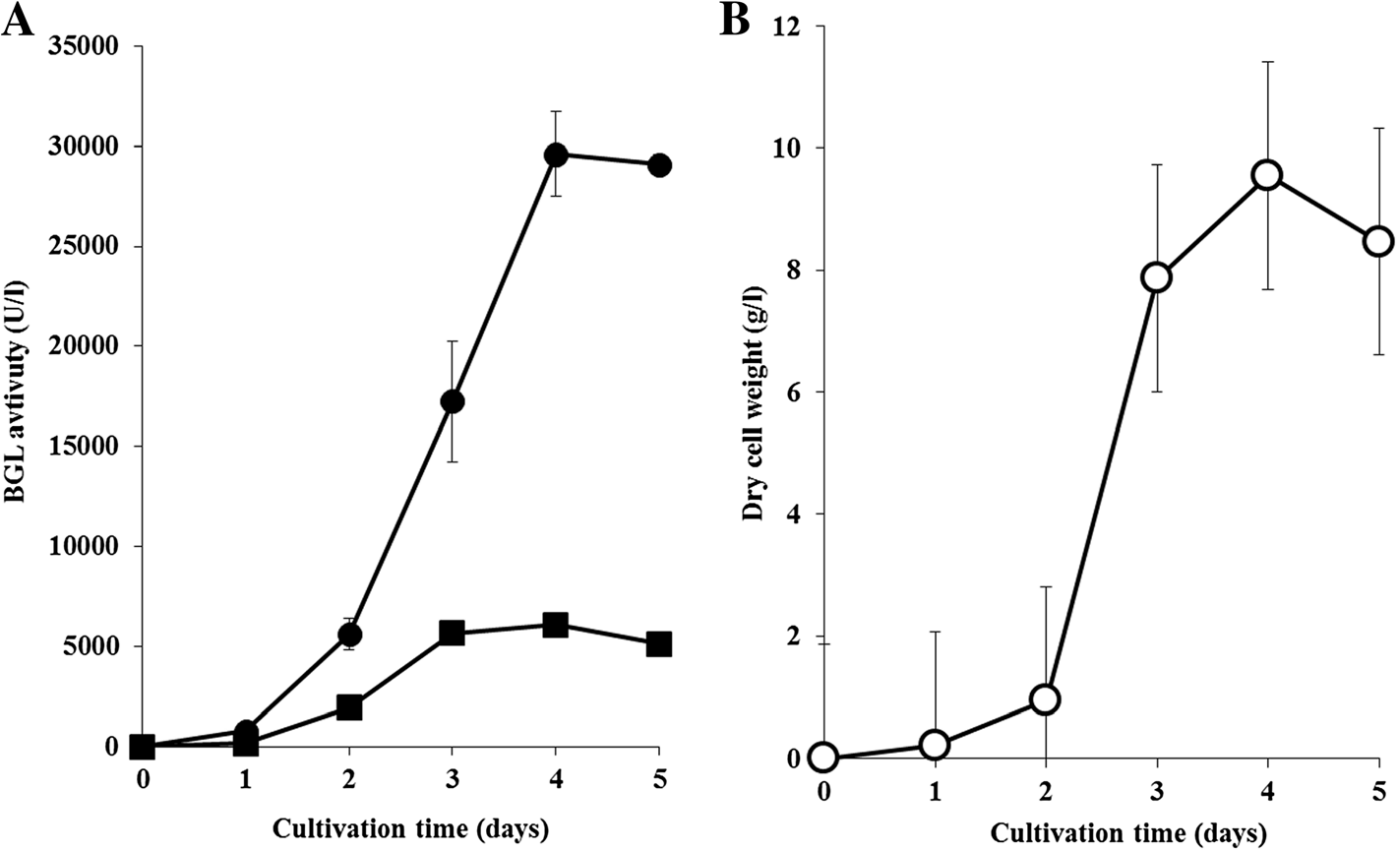
**The secretory production of BGL using two different vectors with XCs. (A)** Time-course of culture supernatant β-glucosidase activity: *S. lividans*/pT-xcs-pU-xcs (closed circles); *S. lividans*/pT-xcs-pU-plds (closed squares) in modified TSB medium with 5% tryptone and 3% glucose. Each data point shows the average of 3 independent experiments, and error bars represent the standard deviation. **(B)** Time-course of dry cell weight: *S. lividans*/pT-xcs-pU-xcs (open circles). Each data point shows the average of 3 independent experiments, and error bars represent the standard deviation.

**Figure 4:**
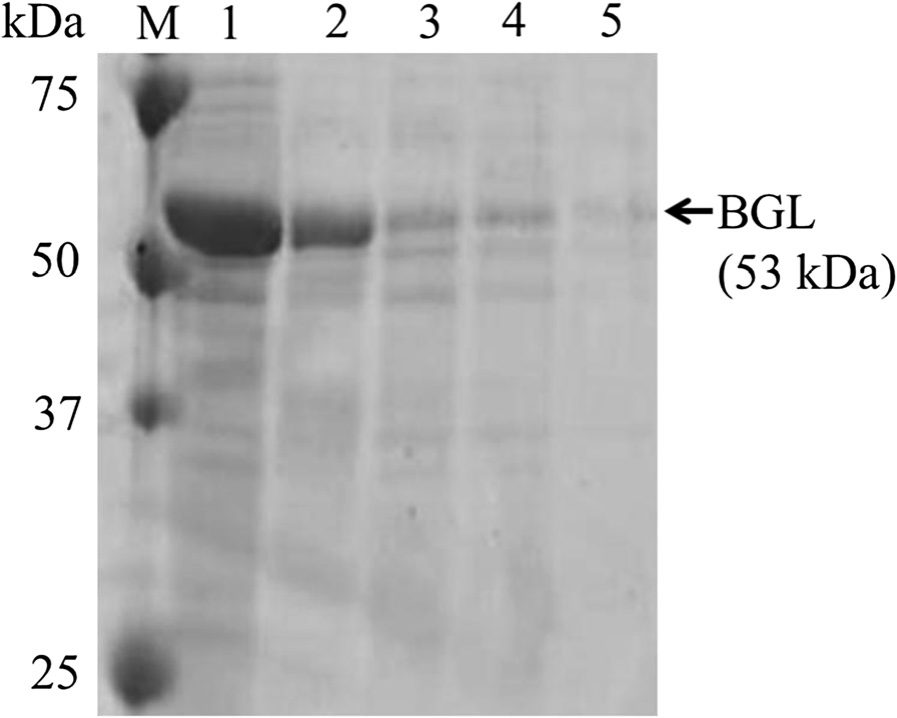
**SDS-PAGE of produced BGL in the culture supernatant of each transformant.** Lane 1: *S. lividans*/pTxcs-pUxcs after 4 days cultivation; Lane 2: *S. lividans*/pU-xcs-bgl after 3 days cultivation; Lane 3: *S. lividans*/pU-0620 s-bgl after 3 days cultivation; Lane 4: *S. lividans*/pU-2486 s-bgl after 3 days cultivation; Lane 5: *S. lividans*/pU-plds-bgl after 3 days cultivation.

In order to achieve further improvement of protein secretion using XCs, we focused on the copy number of BGL genes introduced into *S. lividans*, and introduced the gene encoding BGL into *S. lividans* using two different plasmids, pUC702 and pTYM18. pUC702 is a high-copy number vector derived from pIJ101, whereas pTYM18 is an integrative vector. After creating *S. lividans*/pT-xcs-bgl, we introduced pUC702-xcs-bgl or pUC702-plds-bgl into this transformant, and obtained *S. lividans*/pTxcs-pUxcs or *S. lividans*/pTxcs-pUplds, respectively. Using the two constructed strains, we evaluated the amount of produced BGL. Figure 3(A) shows the time-courses of BGL activity of *S. lividans*/pTxcs-pUxcs and *S. lividans*/pTxcs-pUplds. The maximal level of BGL activity reached 30,000 U/l in the cultivation of *S. lividans*/pTxcs-pUxcs, which was 17 times as much as that of *S. lividans*/pU-plds-bgl. We also evaluated the amount of secreted BGL using *S. lividans*/pT-xcs-bgl. The BGL activity detected in the culture supernatant of *S. lividans*/pT-xcs-bgl was 6,000 U/l, whereas BGL activity in the culture supernatant of *S. lividans*/pU-xcs-bgl was 22,000 U/l. An obvious band corresponding to BGL was detected in SDS-PAGE analysis of the culture supernatant of *S. lividans*/pTxcs-pUxcs, and the amount of produced BGL reached 1,100 mg/l after 4 days cultivation (Figure 4). However, when using *S. lividans*/pTxcs-pUplds, the maximal level of BGL activity was 6,100 U/l, which was less than that when using *S. lividans*/pU-xcs-bgl. Here, we measured the specific activities of BGL produced by *S. lividans*/pU-xcs-bgl and *S. lividans*/pU-plds-bgl, respectively. The specific activity of BGL secreted by XCs was 32.3 U/mg, whereas that of Plds was 37.0 U/mg. Thus, the difference of signal peptides didn’t affect the specific activity of produced BGL.

## Discussion

The goal of this study was to develop a hyper-secretion system for useful proteins using *S. lividans*. In order to achieve this, we focused on signal peptides, which encourage protein secretion, and on the copy number of genes introduced into *S. lividans*. First, candidate signal peptides were screened from biomass-degradation enzymes of *T. fusca* and *S. lividans* in accordance with our hypothesis that signal peptides of biomass-degradation enzymes can enhance the level of secreted proteins. As a result, we successfully obtained 3 signal peptides that promoted protein secretion more efficiently than the previously discovered Plds. Then we tried to improve protein secretion by increasing the copy number of the gene encoding BGL in *S. lividans*. BGL fused to XCs was introduced into *S. lividans* using two kinds of plasmid, pUC702 and pTYM18. As a result, the amount of secreted BGL was drastically increased compared to in the case of using only pUC702 with Plds (Figure 4).

In this study, although 3 signal peptides were chosen from the biomass-degradation enzyme screen in *T. fusca* and *S. lividans*, they were all Tat-dependent signal peptides according to previous reports [19,22]. This result may indicate that Tat-dependent signal peptides are more suitable than Sec-dependent ones in the secretory production of the BGL model we adopted. The Sec system secretes unfolded proteins across the membrane, whereas the Tat system can transport proteins that are fully folded. However, it has been also reported that the export pathway preference is determined by the properties of the mature protein [1]. The BGL we used in this study does not possess a signal peptide and is known to be produced as a non-secreted protein, indicating that BGL is fully folded inside of the cell [24]. Therefore, by using a Tat-dependent signal peptide to secrete BGL, BGL was folded within the cell and efficiently secreted into the culture supernatant. On the other hand, by using a Sec-dependent signal peptide, BGL was folded during or after secretion into the extracellular environment. The mechanism of protein folding in the Tat system is similar to that of native BGL, and therefore Tat-dependent signal peptides might have been isolated more preferentially than Sec-dependent signal peptides. Page et al. previously reported that the amount of secreted xylanase C, which depends on the Tat system, was decreased when the original signal peptides were replaced with Sec-dependent signal peptides [25]. This result corresponds with our present findings.

Protein secretion is encouraged by two different kinds of energy use, ATP hydrolysis and proton motive force (PMF). The Sec system is known to require both ATP hydrolysis and PMF for translocation outside of cells, whereas the Tat system needs only PMF. Vrancken et al. reported on a relationship between protein secretion and phage-shock protein A (PspA), which is supposed to play a role in the maintenance of PMF. In their report, the overexpression of the gene encoding PspA (*pspA*) enhanced the amount of secreted protein when using Tat-dependent signal peptides more so than when using Sec-dependent ones [26]. This indicates that introduction of *pspA* into the created strain in this study may lead to further BGL productivity.

In the present study, we also carried out BGL secretion using two kinds of plasmid in order to increase the copy number of the BGL gene in *S. lividans*. After BGL-secreting *S. lividans* was constructed using pTYM18 involving XCs for BGL secretion, we introduced pUC702 carrying XCs or Plds for BGL secretion into the constructed strain, and created *S. lividans*/pTxcs-pUxcs or *S. lividans*/pTxcs-pUplds. As shown in Figure 3, the maximal level of produced BGL reached 30,000 U/l when using *S. lividans*/pTxcs-pUxcs. The maximal BGL activity in the culture supernatant of *S. lividans*/pT-xcs-bgl or *S. lividans*/pU-xcs-bgl was 6,000 or 21,000 U/l, respectively. These results indicate that both pTYM18-xcs-bgl and pUC702-xcs-bgl were retained in *S. lividans*/pTxcs-pUxcs and that they encouraged BGL secretion. The maximal level of produced BGL in the culture supernatant reached 1,100 mg/l, which is the highest protein secretion productivity using *Streptomyces* as the host strain yet achieved (Figure 4) [1]. Although *S. lividans*/pTxcs-pUxcs has only one or two extra copy of the gene than *S. lividans*/pU-xcs-bgl, BGL productivity was drastically increased. It has been reported that the multi-copy vector doesn’t necessarily encourage protein expression more effectively than the integration vector [27], and our results may be attributed to that. In this study, BGL productivity was enhanced by increasing the copy number of the gene encoding BGL using two kinds of plasmid. The introduction of even more BGL genes has the potential to improve BGL productivity further; however, overexpression of Tat components in *S. lividans* may be more significant. Tat components control the Tat system of *S. lividans* and are composed of TatA, TatB, and TatC [1,12]. De Keersmaeker et al. reported that the amount of produced xylanase C in *S. lividans* increased up to 5 times using the approach of TatABC overexpression [19]. This implies that our current secretion system may be further advanced. Here, in order to confirm the versatility of our secretion system, the secretory production of Tfu0901 (EG), which is one of endoglucanase derived from *T. fusca* YX, was carried out. Although drastic advance was not confirmed in the case of using EG compared to using BGL, EG couldn’t be expressed using the original signal peptide in *S. lividans* and our protein secretory system can effectively encourage protein secretion (data not shown).

In this study, we successfully screened for 3 signal peptides to enhance the level of protein secretion from biomass-degradation enzymes in *T. fusca* and *S. lividans*. Using one of the isolated signal peptides, XCs, the amount of secreted BGL increased up to 10 times as much as that when using Plds, which is a signal peptide we previously isolated. Additionally, by increasing the copy number of BGL genes in *S. lividans* using two kinds of plasmid, we expanded BGL productivity up to 15 times compared to using Plds, and successfully developed a hyper-secretion system that is more efficient than those from previous reports. In order to achieve further protein secretion using our system, we are currently carrying out additional genetic improvements.

## Materials and methods

### Plasmids construction

Each polymerase chain reaction (PCR) was carried out using PrimeSTAR HS (Takara, Shiga, Japan). The plasmids for expressing 25 genes encoding biomass degradation enzymes were constructed, and each gene was expressed in *S. lividans* as Additional file 1. The plasmids for secreting BGL were constructed as follows. The gene fragments encoding each signal peptide were amplified by PCR using the *S. lividans* 1326 (NBRC15675) or *T. fusca* YX genome (ATCC27730) as a template with the corresponding primers (Table 2). The XCs fragment was digested with *Sph*I and *Nhe*I and introduced into the *Sph*I and *Nhe*I sites of pUC702-plds-bgl. Here, pUC702-plds-bgl and *S. lividans*/pU-plds-bgl were previously called pUC702-pro-sig-Tfu0937-(His)_6_-term and *S. lividans*/pUC702-Tfu0937-(His)_6_, respectively [28]. The resultant plasmid was called pUC702-xcs-bgl. The other fragments encoding signal peptides were introduced into the *Sph*I and *Nhe*I sites of pUC702-plds-bgl with the In-Fusion HD Cloning kit (Takara). The resultant plasmids were named as shown in Table 2.

Integration-type vectors for the expression of XCs-BGL and plds-BGL were constructed as follows. pUC702-xcs-bgl and pUC702-plds-bgl were digested with *Hind*III and *Kpn*I, and the digested fragment encoding the promoter, signal sequence, the gene encoding BGL and terminator was subcloned into the *Hind*III and *Kpn*I sites of pTYM18 [23], which is a shuttle vector between *E. coli* and *S. lividans*. The resultant plasmids were called pTYM18-xcs-bgl and pTYM18-plds-bgl, respectively.

### Bacterial strains, transformation, and cultivation

All integration-type plasmids constructed were transformed into *E. coli* S17-1 λpir (Biomedal, Seville, Spain). A single colony of each transformant was picked and cultivated at 37°C for 8 h in 3 mL of LB medium containing 40 μg/mL kanamycin. Cells were then harvested and the cell suspension was washed 3 times with LB broth to remove residual kanamycin. The cells were then suspended in 500 μL of LB broth and mixed with wild-type *S. lividans* 1326 spores. The mixture was plated on ISP4 medium (1.0% soluble starch, 0.1% K_2_HPO_4_, 0.1% MgSO_4_ · 7H_2_O, 0.1% NaCl, 0.2% (NH_4_)_2_SO_4_, 0.2% CaCO_3_, 0.0001% FeSO_4_, 0.0001% MnCl_2_, 0.0001% ZnSO_4_, and 2.0% agar) and incubated for 18 h at 30°C. A 3-mL aliquot of soft-agar nutrient broth containing kanamycin (50 μg/mL) and nalidixic acid (67 μg/mL) was dispensed in layers on the plate, which was then incubated at 30°C for 5–7 days. A single colony was picked and streaked on an ISP4 agar plate containing kanamycin (50 μg/mL) and nalidixic acid (5 μg/mL). The plate was incubated at 30°C for 5–7 days, after which transformants were selected and named as listed in Table 2.

Protoplasts of wild-type *S. lividans* 1326 and *S. lividans*/pT-xcs-bgl were prepared according to the method of Hopwood et al. [29]. Briefly, the mycelium of each strain was treated with a solution of 1 mg/mL lysozyme (Wako, Osaka, Japan), and suspended mycelia were used as protoplasts. Each multi-copy plasmid was introduced into wild-type *S. lividans* 1326 or *S. lividans*/pT-xcs-bgl using the polyethylene glycol (PEG) method. Selection of transformants was carried out by overlaying soft agar containing 50 μg/mL of thiostrepton or 50 μg/mL of thiostrepton and kanamycin. After cultivation for 5 days, transformants were selected and named as listed in Table 2.

For screening candidate signal peptides, spores of each transformant carrying a gene encoding a biomass degradation enzyme or BGL fused to a signal peptide screened in this study were inoculated in a test tube containing 5 ml of TSB medium (17 g/L pancreatic digest of casein, 3 g/L papaic digest of soybean meal, 2.5 g/L glucose, 5.0 g/L sodium chloride, and 2.5 g/L dipotassium phosphate (BD Diagnostic Systems, Sparks, MD, USA)) supplemented with 5 μg/ml of thiostrepton (MP Biomedicals, Illkirch-Graffenstaden, France), followed by cultivation at 28°C for 3 days. In the case of cultivation using *S. lividans*/pTxcs-pUxcs and *S. lividans*/pTxcs-pUplds, 5 μg/ml thiostrepton and 50 μg/ml kanamycin was used as a selective marker. Then, 5 ml of the preculture medium of each transformant was seeded into a shake flask with a baffle containing 100 ml of modified TSB medium with 5 μg/ml thiostrepton, 3% glucose as a carbon source, and 5% tryptone as a nitrogen source, followed by incubation at 28°C for 5 days. In the case of cultivation using *S. lividans*/pTxcs-pUxcs and *S. lividans*/pTxcs-pUplds, 5 μg/ml thiostrepton and 50 μg/ml kanamycin was used as a selective marker.

### Measurement of BGL activity

β-Glucosidase activity was measured in 25 μl of 1 M sodium acetate (pH 7.0) with 100 μl of 10 mM *p*-nitrophenyl-β-D-glucopyranoside (pNPG) (Nacalai Tesque) as the substrate. The mixture (containing 375 μl of culture supernatant diluted to 10–0.01%) was incubated at 50°C for 40 min. The reaction was terminated by the addition of 500 μl of 3 M sodium carbonate, and the *p*-nitrophenol released was determined by measuring absorbance at 400 nm. One unit of enzyme activity was defined as the amount of enzyme that released 1 μmol of *p*-nitrophenol from the substrate per min.

### SDS-polyacrylamide gel electrophoresis (SDS-PAGE) analysis

Culture supernatants of each transformant were directly mixed with SDS-PAGE buffer (2% SDS, 10% glycerol, 5% 2-mercaptoethanol, 0.002% bromophenol blue, 0.125 M Tris–HCl, pH 6.8) and boiled. The protein samples were fractionated by a 15% SDS-PAGE gel and stained with Coomassie Brilliant Blue R-250 (Nacalai Tesque).

### Western blotting analysis

Sodium dodecyl sulfate-polyacrylamide gel electrophoresis (SDS-PAGE) loading buffer was added to the supernatant of each transformant followed by boiling at 100°C for 5 min. Proteins were analyzed by SDS-PAGE using an SDS-polyacrylamide gel (15%: w/v), after which proteins were electroblotted onto a polyvinylidene difluoride membrane (Millipore Co., Boston, MA, USA) and were allowed to react with primary rabbit anti-(His)_6_ and secondary goat anti-rabbit immunoglobulin G alkaline phosphatase conjugated antibodies (Promega Co., Madison, WI, USA). The membrane was then stained with nitroblue tetrazolium (Promega) and 5-bromo-4-chloro-3-indolylphosphate (Promega) according to the manufacturer’s protocol.

### Measurement of secreted BGL in the culture supernatant

After SDS-PAGE, the concentration of secreted BGL in the culture supernatant was evaluated with ImageQuant TL (GE Healthcare, Tokyo, Japan) using purified BGL as a standard. The concentration of purified BGL was quantified using a Quick Start Bradford Protein Assay (BioRad Laboratories, Hercules, CA).

## Competing interests

The authors declare that they have no competing interests.

## Authors’ contributions

TM and SN designed the experiments. TM performed the experiments. TM, SN and TT wrote the paper. AK commented and supervised on the manuscript. All authors approved the final manuscript.

## Supplementary Material

Additional file 1 **Additional materials and methods.** Plasmids construction and expression of biomass degradation enzymes. Click here for file
