# Supplementary material for: Hyper secretion of Thermobifida fusca β-glucosidase via a Tat-dependent signal peptide using Streptomyces lividans
Source: Microb Cell Fact. 2013 Oct 1;12:88. doi: 10.1186/1475-2859-12-88 (PMC3850917; doi:10.1186/1475-2859-12-88)
Supplement: Additional file 1 — Additional materials and methods. Plasmids construction and expression of biomass degradation enzymes. [file 1475-2859-12-88-S1.docx]

**Additional Materials and methods**

**Plasmids construction and expression of biomass degradation enzymes**

Each polymerase chain reaction (PCR) was carried out using PrimeSTAR HS (Takara). The plasmids for expressing 23 genes encoding biomass degradation enzymes were constructed as Additional file 1. The gene fragments encoding each biomass degradation enzyme derived were amplified by PCR using the *S. lividans* 1326 (NBRC15675) or *T. fusca* YX genome (ATCC27730) as a template with the corresponding primers (Table S1). The all fragments encoding signal peptides were introduced into the *Sph*I and *Nhe*I sites of pUC702-pro-sig-term with the In-Fusion HD Cloning kit (Takara). The resultant plasmids were named as shown in Table S1.

Each plasmid was introduced into wild-type *S. lividans* 1326 using the polyethylene glycol (PEG) method. Selection of transformants was carried out by overlaying soft agar containing 50 μg/mL of thiostrepton. After cultivation for 5 days, transformants were selected and named as listed in Table 2.

Spores of each transformant carrying a gene encoding a biomass degradation enzyme were inoculated in a test tube containing 5 ml of TSB medium supplemented with 5 μg/ml of thiostrepton (MP Biomedicals, Illkirch-Graffenstaden, France), followed by cultivation at 28°C for 3 days. The culture supernatant of each transformant was applied to western blotting analysis in order to confirm whether each biomass degradation enzyme was expressed.

Table S1. Plasmids, transformants, and oligonucleotide primers used in this study.

| Plasmid, primer, | Relevant features | Source or reference |
| --- | --- | --- |
| or transformant |  |  |
| Plasmids |  |  |
| pUC702-xa | Vector for secreting xylanase A; thiostrepton resistance marker | This study |
| pUC702-xb | Vector for secreting xylanase B; thiostrepton resistance marker | This study |
| pUC702-xc | Vector for secreting xylanase C; thiostrepton resistance marker | This study |
| pUC702-cb | Vector for secreting cellulase B; thiostrepton resistance marker | This study |
| pCU702-tfu0620 | Vector for secreting Tfu0620; thiostrepton resistance marker | This study |
| pUC702-tfu2486 | Vector for secreting Tfu2486; thiostrepton resistance marker | This study |
| pUC702-tfu2176 | Vector for secreting Tfu2716; thiostrepton resistance marker | This study |
| pUC702-tfu1612 | Vector for secreting Tfu1612; thiostrepton resistance marker | This study |
| pUC702-tfu2788 | Vector for secreting Tfu2788; thiostrepton resistance marker | This study |
| pUC702-tfu0082 | Vector for secreting Tfu0082; thiostrepton resistance marker | This study |
| pUC702-tfu0153 | Vector for secreting Tfu0153; thiostrepton resistance marker | This study |
| pUC702-tfu0868 | Vector for secreting Tfu0868; thiostrepton resistance marker | This study |
| pUC702-tfu0900 | Vector for secreting Tfu0900; thiostrepton resistance marker | This study |
| pUC702-tfu0985 | Vector for secreting Tfu0985; thiostrepton resistance marker | This study |
| pUC702-tfu1213 | Vector for secreting Tfu1213; thiostrepton resistance marker | This study |
| pUC702-tfu2168 | Vector for secreting Tfu2168; thiostrepton resistance marker | This study |
| pUC702-tfu2712 | Vector for secreting Tfu2712; thiostrepton resistance marker | This study |
| pUC702-tfu2791 | Vector for secreting Tfu2791; thiostrepton resistance marker | This study |
| pUC702-tfu1268 | Vector for secreting Tfu1268; thiostrepton resistance marker | This study |
| pUC702-tfu1616 | Vector for secreting Tfu1616; thiostrepton resistance marker | This study |
| pUC702-tfu1621 | Vector for secreting Tfu1621; thiostrepton resistance marker | This study |
| pUC702-tfu1629 | Vector for secreting Tfu1629; thiostrepton resistance marker | This study |
| pUC702-tfu1665 | Vector for secreting Tfu1665; thiostrepton resistance marker | This study |
| pUC702-tfu2789 | Vector for secreting Tfu2789; thiostrepton resistance marker | This study |
| pUC702-tfu2990 | Vector for secreting Tfu2990; thiostrepton resistance marker | This study |
|  |  |  |
| Transformants |  |  |
| *S. lividans*/pU-xa | Transformant harboring pUC702-xa | This study |
| *S. lividans*/pU-xb | Transformant harboring pUC702-xb | This study |
| *S. lividans*/pU-xc | Transformant harboring pUC702-xc | This study |
| *S. lividans*/pU-cb | Transformant harboring pUC702-cb | This study |
| *S. lividans*/pU-0620 | Transformant harboring pUC702-tfu0620 | This study |
| *S. lividans*/pU-2486 | Transformant harboring pUC702-tfu2486 | This study |
| *S. lividans*/pU-2176 | Transformant harboring pUC702-tfu2176 | This study |
| *S. lividans*/pU-1612 | Transformant harboring pUC702-tfu1612 | This study |
| *S. lividans*/pU-2788 | Transformant harboring pUC702-tfu2788 | This study |
| *S. lividans*/pU-0082 | Transformant harboring pUC702-tfu0082 | This study |
| *S. lividans*/pU-0153 | Transformant harboring pUC702-tfu0153 | This study |
| *S. lividans*/pU-0868 | Transformant harboring pUC702-tfu0868 | This study |
| *S. lividans*/pU-0900 | Transformant harboring pUC702-tfu0900 | This study |
| *S. lividans*/pU-0985 | Transformant harboring pUC702-tfu0985 | This study |
| *S. lividans*/pU-1213 | Transformant harboring pUC702-tfu1213 | This study |
| *S. lividans*/pU-2168 | Transformant harboring pUC702-tfu2168 | This study |
| *S. lividans*/pU-2712 | Transformant harboring pUC702-tfu2712 | This study |
| *S. lividans*/pU-2791 | Transformant harboring pUC702-tfu2791 | This study |
| *S. lividans*/pU-1268 | Transformant harboring pUC702-tfu1268 | This study |
| *S. lividans*/pU-1616 | Transformant harboring pUC702-tfu1616 | This study |
| *S. lividans*/pU-1621 | Transformant harboring pUC702-tfu1621 | This study |
| *S. lividans*/pU-1629 | Transformant harboring pUC702-tfu1629 | This study |
| *S. lividans*/pU-1665 | Transformant harboring pUC702-tfu1665 | This study |
| *S. lividans*/pU-2789 | Transformant harboring pUC702-tfu2789 | This study |
| *S. lividans*/pU-2990 | Transformant harboring pUC702-tfu2990 | This study |
|  |  |  |
| Oligonucleotide primers |  |  |
| xa_Fw | TCGTTTAAGGATGCAatgggctcctacgcccttcc |  |
| xa_Rv | CGATTGCGAGGTCACgtggtggtggtggtggtgggtgcgggtccagcgttggt |  |
| xb_Fw | TCGTTTAAGGATGCAatgaacctgctcgtccagcc |  |
| xb_Rv | CGATTGCGAGGTCACgtggtggtggtggtggtggcccgcgctgcaggacacgc |  |
| xc_Fw | TCGTTTAAGGATGCAatgcagcaggacggcacacagcagg |  |
| xc_Rv | CGATTGCGAGGTCACgtggtggtggtggtggtgggcgtgggctgtgccgggcagcagc |  |
| cb_Fw | TCGTTTAAGGATGCAatgcgaacgttacggcccca |  |
| cb_Rv | CGATTGCGAGGTCACgtggtggtggtggtggtgcaccgtggtgcaggcggtgc |  |
| tfu0620_Fw | TCGTTTAAGGATGCAatgagtaaagttcgtgccacgaaca |  |
| tfu0620_Rv | CGATTGCGAGGTCACgtggtggtggtggtggtgggcggcgttggccggagcagcgaac |  |
| tfu2486_Fw | TCGTTTAAGGATGCAatgtttcgacgtctgcctgtgctgg |  |
| tfu2486_Rv | CGATTGCGAGGTCACgtggtggtggtggtggtgtgccgcctgagcgtccacgtcagcg |  |
| tfu2176_Fw | TCGTTTAAGGATGCAatgtccgtcactgaacctcctcccc |  |
| tfu2176_Rv | CGATTGCGAGGTCACgtggtggtggtggtggtgttcggcgtgggcggttcccgtggcc |  |
| tfu1612_Fw | TCGTTTAAGGATGCAatgacagcaacagcacagcgaacac |  |
| tfu1612_Rv | CGATTGCGAGGTCACgtggtggtggtggtggtgggcggcagcggagtggacgaggccg |  |
| Tfu2788_Fw | TCGTTTAAGGATGCAatgtccgtcactgaacctcctcccc |  |
| Tfu2788_Rv | CGATTGCGAGGTCACgtggtggtggtggtggtgttcggcgtgggcggttcccgtggcc |  |
| Tfu0082_Fw | TCGTTTAAGGATGCAatgctgcacctgacccgacg |  |
| Tfu0082_Rv | CGATTGCGAGGTCACgtggtggtggtggtggtggcatccggatacgcgctccc |  |
| Tfu0153_Fw | TCGTTTAAGGATGCAatgcgaagagctgccaccct |  |
| Tfu0153_Rv | CGATTGCGAGGTCACgtggtggtggtggtggtggaggatgtggcccaccccgg |  |
| Tfu0868_Fw | TCGTTTAAGGATGCAatggcgggtcgacaccggat |  |
| Tfu0868_Rv | CGATTGCGAGGTCACgtggtggtggtggtggtggcgcagcgactccgccatcg |  |
| Tfu0900_Fw | TCGTTTAAGGATGCAatgagaaaacgtctcgcggt |  |
| Tfu0900_Rv | CGATTGCGAGGTCACgtggtggtggtggtggtggcgagcggtgcagctcagcg |  |
| Tfu0985_Fw | TCGTTTAAGGATGCAatgggagtgcgcagatccct |  |
| Tfu0985_Rv | CGATTGCGAGGTCACgtggtggtggtggtggtggcgccaggagtcgtagaagt |  |
| Tfu1213_Fw | TCGTTTAAGGATGCAatgaaccatgcccccgccag |  |
| Tfu1213_Rv | CGATTGCGAGGTCACgtggtggtggtggtggtggttggcgctgcaggacaccg |  |
| Tfu2168_Fw | TCGTTTAAGGATGCAatgggacgatcaattacgcg |  |
| Tfu2168_Rv | CGATTGCGAGGTCACgtggtggtggtggtggtggccggcgtaggtttcgaact |  |
| Tfu2712_Fw | TCGTTTAAGGATGCAatgggcagaatgacccccct |  |
| Tfu2712_Rv | CGATTGCGAGGTCACgtggtggtggtggtggtgggcgggaggcgtaccccaga |  |
| Tfu2791_Fw | TCGTTTAAGGATGCAatgaaaccggtgcgtctcat |  |
| Tfu2791_Rv | CGATTGCGAGGTCACgtggtggtggtggtggtggcagtgatcgtgcttggggc |  |
| Tfu1268_Fw | TCGTTTAAGGATGCAatgcatcgttactctcgtac |  |
| Tfu1268_Rv | CGATTGCGAGGTCACgtggtggtggtggtggtggacgaagttcacgtcgctgc |  |
| Tfu1616_Fw | TCGTTTAAGGATGCAatgacgtctccccaagtcac |  |
| Tfu1616_Rv | CGATTGCGAGGTCACgtggtggtggtggtggtgggagggggactgaggccggt |  |
| Tfu1621_Fw | TCGTTTAAGGATGCAatggtgtggaaaacctggaa |  |
| Tfu1621_Rv | CGATTGCGAGGTCACgtggtggtggtggtggtggtcgcgccgagtgtaagcca |  |
| Tfu1629_Fw | TCGTTTAAGGATGCAatgagcaacgaccacctccc |  |
| Tfu1629_Rv | CGATTGCGAGGTCACgtggtggtggtggtggtggccgcggtggcgggcgcggt |  |
| Tfu1665_Fw | TCGTTTAAGGATGCAatgagaatgagaagccggct |  |
| Tfu1665_Rv | CGATTGCGAGGTCACgtggtggtggtggtggtgggccacggagcagaggctgc |  |
| Tfu2789_Fw | TCGTTTAAGGATGCAatgctctgtgggcaaacgaa |  |
| Tfu2789_Rv | CGATTGCGAGGTCACgtggtggtggtggtggtggcccagacgcaggacgatca |  |
| Tfu2990_Fw | TCGTTTAAGGATGCAatgtcccgtacctgggcacg |  |
| Tfu2990_Rv | CGATTGCGAGGTCACgtggtggtggtggtggtggtcaagcagcgggatcagcg |  |
